# Survey of Spanish pet owners about endoparasite infection risk and deworming frequencies

**DOI:** 10.1186/s13071-020-3976-8

**Published:** 2020-02-26

**Authors:** Guadalupe Miró, Rosa Gálvez, Ana Montoya, Beatriz Delgado, Jason Drake

**Affiliations:** 1 Animal Health Department, Veterinary Faculty, Universidad Complutense de Madrid, Avda. Puerta de Hierro s/n, 28040 Madrid, Spain; 2 Elanco Spain, Avda. de la Industria, 30-28108 Alcobendas, Spain; 3 Elanco, 2500 Innovation Way, Greenfield, IN 46140 USA

**Keywords:** ESCCAP, Risk assessment, Intestinal parasites, Zoonosis, Parasite control, Dogs, Cats, Spain

## Abstract

**Background:**

Pets may be carriers of infectious agents including parasites. As part of a larger-scale study covering the whole of Europe, this study examines deworming measures reported by Spanish pet owners and identifies risk factors.

**Methods:**

An online questionnaire was administered to cat and dog owners in Spain. The replies provided were used to obtain information about the petsʼ living conditions and to accordingly classify each pet into one of the four ESCCAP infection risk categories (A, B, C or D) for which different deworming frequencies are recommended. Questions were also asked about pet care and ownersʼ attitude toward their pets. The Kruskal-Wallis test was used to correlate risk groups with deworming frequencies.

**Results:**

Completed questionnaires were returned by 500 cat owners and 501 dog owners. According to responses, 96.21% of dogs were assigned to risk category D (maximum risk), and only 1.2%, 2.2% and 0.4% to A, B and C, respectively. Almost all cats were assigned to the minimum risk category A (indoor cats, 62%) or maximum risk category D (outdoor cats, 32.8%); only 3.4% and 1.8% of cats were classified as risk B and C respectively. More dogs were allocated to the higher risk group compared to cats, which were more frequently kept indoors. Cats were reportedly dewormed less frequently than dogs (2.56 and 3.13 times per year respectively), consistent with their different infestation risk. Thus, pets in the lower risk group A were either adequately dewormed or treated more often than necessary. Only a small proportion of cats were not dewormed at all (*n* = 14). Alarmingly, almost all pets in risk groups B, C or D (representing 95% of dogs and 39% of cats) were dewormed less often than recommended.

**Conclusions:**

More effective health education is required for the management of zoonotic endoparasite diseases under the umbrella of One Health targeted at owners, veterinarians, general practitioners, and health authorities. To align deworming frequency with infection risk, pet owners should be provided with clear, compelling instructions.
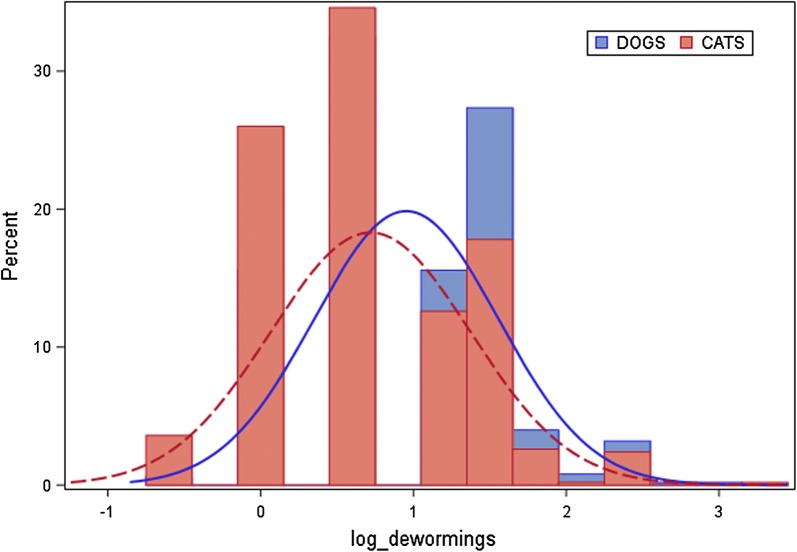

## Background

According to European data for 2018, the number of Spanish homes with at least one pet was 39.7% and there were 6,270,000 dogs [1] and 3,145,000 cats [2] living in homes across Spain. Although pets offer significant psychological and physical benefits for their owners [3], there are also well-documented health hazards associated with owning a pet including bites, scratches and allergies. Pets can also be carriers of infectious agents (such as parasites, bacteria, fungi and viruses) despite appearing to be healthy [4]. More importantly, a wide range of parasites affecting pets have zoonotic potential, mainly members of the groups protozoa, helminths and arthropods. Helminths, including nematodes, cestodes and trematodes, commonly infect dogs and cats in Europe [4]. Prevalent parasites with significant zoonotic potential are the intestinal worms: *Toxocara* spp. (family Ancylostomatidae), *Dipylidium caninum* and species of the family Taenidae (genera *Taenia* and *Echinococcus*) [4].

*Toxocara canis* and *T. cati* are cosmopolitan parasites. In Spain, the prevalence of these parasite infections varies between 7.4–31.8% in dogs and 7.7–58.0% in cats [5–11]. However, prevalence data from different studies are difficult to compare due to differences in age (prevalence is higher in puppies and kittens), habitat (e.g. shelters or refuges, stray animals), and diagnostic techniques (coprological technique, *post-mortem*, etc.) [5–11]. *Toxocara* spp. are also among the parasites found most frequently in playground sand, and prevalences in soil samples from public parks in Spain have been estimated at 3.8–16.4% [12, 13]. These parasites may have a significant impact on public health, particularly that of children who use playgrounds frequently as *Toxocara* spp. may cause visceral or ocular larva migrans in humans [14–16].

Roundworms of the family Ancylostomatidae (*Ancylostoma* spp. and *Uncinaria stenocephala*) are responsible for cutaneous larva migrans in humans, ranging in prevalence between 4.3–25.7% and 3.0–91.0% in owned and stray dogs and cats in Spain, respectively [5–8, 10, 11]. These parasites have been also detected in soil samples (3.0–9.3%) in parks in Madrid [12]. This finding is worrying as *Ancylostoma* spp. eggs hatch in the soil and then moult several times within a week developing into infective larvae. While oral ingestion of these larvae is the most common transmission route, they are also able to penetrate the skin of humans. Thus, humans may experience itching due to the movement of larvae, and secondary bacterial infections acquired through scratching are common. In massive infections, the larvae may penetrate into deeper tissues, leading to pulmonary or intestinal symptoms [4].

Among the tapeworms, *D. caninum* infestations are common in Spain and affect 8.3–39.0% of dogs and 4.2–64.6% of cats [6–11, 17]. It is unusual for human adults to be affected as transmission occurs through the inadvertent ingestion of fleas or lice infected with the cysticercoid, and dipylidiosis is more often found in young children. The risk of infection by *D. caninum* can be reduced by effective control of lice and fleas and by regular treatment of pets with a cestocide such as praziquantel [18].

The family Taenidae, which includes the genera *Taenia* and *Echinococcus*, is responsible for other tapeworm infestations commonly found in pets [19]. Infections of humans with metacestodes of different carnivore-specific *Taenia* spp. are rare, although some cases of coenurosis caused by *Taenia multiceps* and *T. serialis*, and of cysticercosis caused by *T. crassiceps* and *T. martis* have been described [20]. Cystic and alveolar echinococcosis caused by *Echinococcus granulosus* and *E. multilocularis,* respectively, are considered among the most serious helminth zoonoses due to their high pathogenic potential [18, 20]. *Echinococcus multilocularis* has been described as an emerging public health threat as urban foci of infection have appeared in some European countries (e.g. Switzerland) [19, 21, 22]). So far, however, infections by adults or metacestodes of *E. multilocularis* have not been reported in Spain, *E. granulosus* remaining as the main cestode responsible for cystic echinococcosis in humans in this country [23].

Because of urban areas with large numbers of pets and limited open spaces, dogs often concentrate in public places such as beaches, parks and playgrounds. These sites can be contaminated with their faeces, making them significant risk areas for the transmission of parasites that affect humans [24]. Unlike the eggs of *Toxocara* spp. and *Ancylostoma* spp., those of *Echinococcus* spp. and *Taenia* spp. are immediately infective upon passage. So, humans (mainly children) may become infected when playing in contaminated playgrounds or playing with dogs, as eggs adhere to hairs around the infected dog’s anus, muzzle and paws [18, 25]. Currently, however, the main source of tapeworm infection in humans appears to be the ingestion of contaminated vegetables and fruits [26, 27].

According to ESCCAP guidelines, pet owners should follow a set of simple preventive measures (e.g. do not eat/give their pets raw meat, wash their hands before eating, wash items and surfaces that have been in contact with raw food, use gloves when gardening, properly wash and disinfect fruit and vegetables and daily remove pet faeces from the environment). Additionally, pets should be seen by a veterinarian to assess their health state and undergo coprological examination at least twice per year. According to coprological results and living conditions, they need deworming on a regular basis [22, 25, 28].

Moreover, these guidelines recommend a deworming regimen designed specifically for each pet based upon an individual assessment of risk factors (age, reproductive status, health status, nutrition, shared accommodation, roaming, working dogs, location and travel history) [28]. Routine deworming procedures should be recommended by practitioners taking into account local epidemiological circumstances and these individual risk factors. In some countries or regions, deworming of cats and dogs is required for health reasons by law. In some Spanish regions, deworming against tapeworms at least once a year is mandatory. This frequency, however, has been described as insufficient for effective control of echinococcosis [19, 29].

Education is the key to controlling certain zoonoses. Veterinarians are often obliged to inform pet owners about possible risks and offer detailed information about parasite transmission routes, importance of deworming from a public health perspective and the protection of other pets, as well as the preventive measures that will help owners and pets to remain healthy. Activities such as the daily removal of faeces from the environment will reduce the likelihood of infection in both pets and owners [18, 28].

According to the ESCCAP, there is also an important risk of importing disease when introducing dogs from abroad. For example, *E. multilocularis* is endemic in some European countries, while it has never been detected in Spain. This means that imported dogs should be checked for these zoonotic diseases and be dewormed correctly by a veterinarian as these parasites can cause long-term health problems in both humans and pets [19, 22].

Zoonotic intestinal parasites are not the only concern, as other worms can cause severe disease in dogs and cats and some are also causes of zoonoses like the heartworms *Dirofilaria immitis*, *Dirofilaria repens* and *Thelazia callipaeda*, or lungworms (*Angiostrongylus vasorum*, *Crenosoma vulpis*, etc.) [30]. It is therefore important that pets are examined by a veterinarian who will accordingly make the relevant recommendations for parasite control.

There is scarce information available about the endoparasite infection risk of dogs and cats in Spain. This risk is related to the animalʼs living conditions including geographical area, travel history, diet, etc. While several studies have determined the prevalence of endoparasites in dog and cat populations, these data often refer to stray populations and cannot be extrapolated to owned pets so it is difficult for the veterinarian to assess the real infection risk for dogs and cats without a thorough anamnesis. This information is extremely important to make decisions about deworming schedules. The objective of the present survey was thus to collect self-reported data from the owners of dogs and cats and to classify pets using these data into the four different infection risk categories defined in the ESCCAP guidelines. After identifying the main risk factors for individual animals, we assessed whether current deworming habits complied with ESCCAP guidelines for the control of tapeworms and roundworms.

## Methods

### Study design

Cat and dog owners in five European countries completed an online questionnaire from July 3, 2017 to July 14, 2017. A description of this survey has been published elsewhere [31]. The data used for the present study were collected in Spain. Questionnaire responses were anonymous and confidential. When cats and dogs lived in the same home, respondents were randomly assigned to either the cat or dog group. The inclusion criteria for respondents owning at least one cat and/or one dog were: (i) age 18 years or older; (ii) being the person mainly responsible for the pet’s health care, feeding and visits to the vet; (iii) taking their pet to the vet at least once a year. To ensure responders treated their pets as companion animals, exclusion criteria were: (i) breeder or trader; (ii) owning more than ten cats or dogs; and (iii) professional use of animal.

Seventeen screening questions ensured owner eligibility (fulfilment of inclusion criteria). Quotas were set according to Spanish owner demographic characteristics to ensure a representative sample of the target population. These characteristics were: age, place where they lived (rural area, town, suburban area, city), living in a household, children living in household, employment status and gender.

The main survey consisted of 7 or 9 questions designed for cat or dog owners respectively. To avoid the risk of answers conditioned by the subsequent set of questions, the first question asked was: How often is your pet dewormed within a year? The following questions were designed to obtain information about the pets living conditions (e.g. outdoor sleeping, hunting habits, feeding habits, living with children/elderly persons). The responses given were interpreted to classify each pet into one of the four different risk groups (A, B, C or D) based upon risks described in the Spanish ESCCAP guidelines, for which different deworming frequencies are recommended (see Table 1 for a detailed description).

**Table 1:** ESCCAP parasite infection risk group definitions and deworming recommendations for pets without considering special risk factors (puppies, kittens, animals used for exhibitions) after Strube et al. [32]

Risk group	Description	Deworming frequency against roundworms and tapeworms
A	Lives indoors only or goes outdoors but has no direct contact with dogs and cats of other households and does not eat prey animals/raw meat, carrion or faeces	1–2 times per year
B	Goes outdoors under supervision and has direct contact with dogs and cats of other households but does not eat prey animals/raw meat, carrion or faeces	4 times per year
C	Goes outdoors under supervision and has direct contact with dogs and cats of other households and eats prey animals/raw meat, but does not eat carrion or faeces	4 times per year against roundworms, 12 times per year against tapeworms
D	Goes outdoors without supervision or under supervision and has direct contact with dogs and cats of other households and eats carrion or faeces	12 times per year

The final set of questions was related to owner opinions about deworming products, their relationship with their pet, satisfaction with their veterinarian, and knowledge about deworming products and source of information.

### Statistical analysis

Distributions of the quantitative variables are provided as means and standard deviations. Categorical variables are expressed as percentages. The Kruskal-Wallis test was used to assess risk category and deworming frequency of pets according to variables recorded in the questionnaire. All statistical tests were performed using the SPSS 25 package (SPSS Inc., Chicago, IL, USA). Significance was set at *P* < 0.05.

## Results

Of 35,830 people invited to participate by email, the entry page was visited by 3,173. Of these, 145 failed to complete the survey, 1,408 did not meet the inclusion criteria and 619 were excluded as the quota had been exceeded [31]. The remaining respondents providing data for the present study were 501 dog owners and 500 cat owners living in Spain. Each respondent provided information about one dog or one cat.

The data collected from the dog and cat questionnaires (501 dogs, 500 cats) are provided in Tables 2 and 3 and are related to pet care and living conditions, respectively.

**Table 2:** Results of the dog and cat questionnaires. Variables related to owners

Variable	Dog dataset (*n* = 501)	Cat dataset (*n* = 500)
Sex, *n* (%)	291 (58.08) women	301 (60.20) women
	210 (41.92) men	199 (39.80) men
Mean age ± SD (range) (years)	43.18 ± 11.53 (18–83)	42.72 ± 11.11 (19–78)
Mean annual deworming frequency ± SD (range)	3.13 ± 2.27 (0–20)	2.56 ± 2.17 (0–24)
Attitude toward pets, *n* (%)	247 (49.30) affectionate	192 (38.40) affectionate
	144 (28.74) devoted	179 (35.80) devoted
	40 (7.98) dispassionate	62 (12.40) dispassionate
	70 (13.97) sceptical	67 (13.40) sceptical
Mean no. of people in household ± SD (range)	3.02 ± 1.16 (1–7)	3.00 ± 1.12 (1–7)
Mean no. children in household ± SD (range)	0.66 ± 0.85 (0–4)	0.67 ± 0.85 (0–3)
Responsibility for pet, *n* (%)	244 (48.70) sole	255 (51.00) sole
	257 (51.30) shared	245 (49.00) shared
Vet visits per year, *n* (%)	344 (68.66) multiple	261 (52.20) multiple
	157 (31.34) single	239 (47.80) single

*Abbreviation*: SD, standard deviation

**Table 3:** Results of dog and cat questionnaires. Variables related to pets

Variable	Dog dataset (*n* = 501)	Cat dataset (*n* = 500)
Animal < six months of age, *n*/*N* (%)	19/501 (3.79)	44/500 (8.80)
Interacts with children/elderly, *n*/*N* (%)	454/501 (90.62)	395/500 (79.00)
Contact with other dogs/animals, *n*/*N* (%)	377/501 (75.25)	na
Outdoor access limited to garden, *n*/*N* (%)	102/501 (20.36)	na
Only indoors, *n*/*N* (%)	na	354/500 (70.80)
Off lead, *n*/*N* (%)	79/399 (19.8) ^a^	na
Eats slugs/snails, *n*/*N* (%)	164/501 (32.73)	na
Hunts, *n*/*N* (%)	na	92/146 (63.01)^b^
Catches prey, *n*/*N* (%)	68/501 (13.57)	93/146 (63.70)^b^
Eats raw meat, *n*/*N* (%)	23/433 (5.31) ^c^	24/407 (5.90)^c^
Mean no. dogs in household ± SD (range)	1.33 ± 0.79 (1–10)	na
Mean no. cats in household ± SD (range)	na	1.54 ± 0.96 (1–8)
Cats in household, *n*/*N* (%)	19/501 (3.79)	na
Dogs in household, *n*/*N* (%)	na	216/500 (43.20)
Area of residence, *N* (%)	56 (11.18) rural	64 (12.80) rural
	134 (26.75) town	117 (23.40) town
	94 (18.76) suburban	85 (17.00) suburban
	217 (43.31) city	234 (46.80) city

^a^Of dogs whose outdoor access is not restricted to garden

^b^Of cats allowed to go outdoors

^c^Of pets that do not catch prey or are not allowed to go outside

*Abbreviation*: n, number; N, total number; na, not applicable (dog only or cat only questions, respectively)

### Dogs

Only 3.79% (19/501) of the dogs were under 6 months of age, 90.62% of dogs had contact with children or elderly persons and 75.25% had contact with other dogs or animals. In only 20.36% (102/501), outdoor access was restricted only to their garden and these dogs had no contact with public places (such as parks, sandpits, playgrounds). Of the remaining dogs, 19.8% were allowed off-lead, 32.73% reportedly ate slugs, snails, grass or dug in the garden and 13.57% caught animals such as rabbits or mice, or scavenged carcasses. Of the dogs that did not catch prey animals, 5.31% ate raw meat. Details are reported in Table 3.

**Fig. 1:**
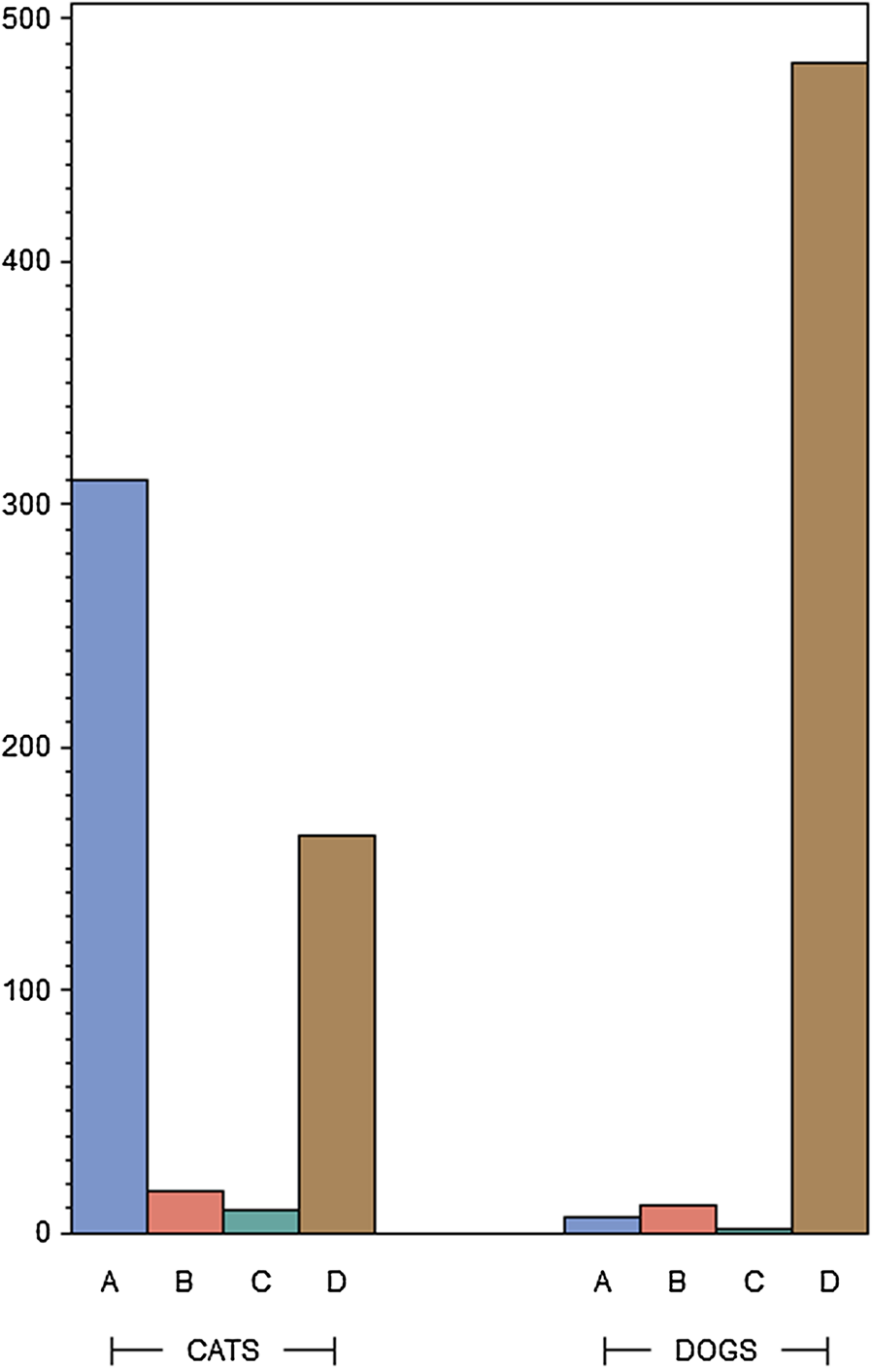
Allocations of dogs and cats to ESCCAP parasite infection risk groups

**Fig. 2:**
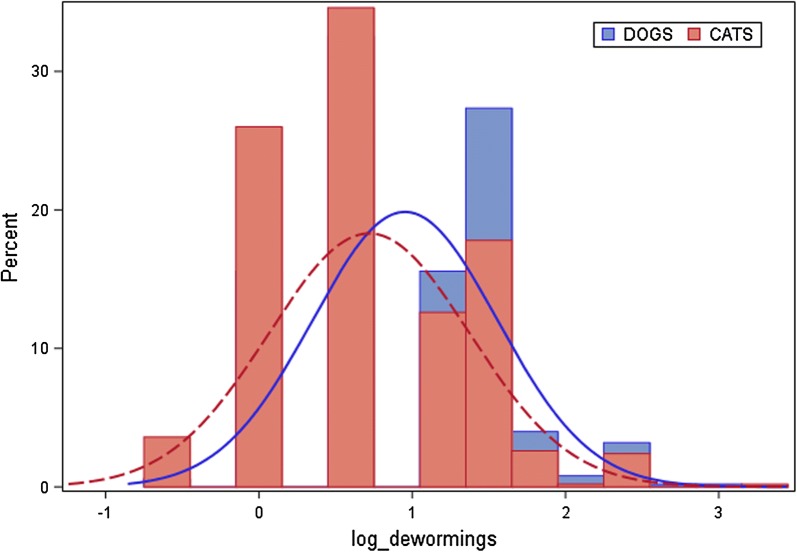
Logarithmically-scaled annual deworming frequencies recorded in the dogs and cats

According to Spanish ESCCAP guidelines, 96.21% (482/501) of the dogs were assigned to risk group D indicating the maximum risk of parasite infection. Only 1.2% (6/501), 2.2% (11/501) and 0.4% (2/501) were assigned to groups A, B and C, respectively [31] (Fig. 1). According to owners, the mean annual deworming frequency (± standard deviation, SD) in dogs was 3.13 ± 2.27 (Fig. 2). By risk group, these frequencies were 1.3, 3.1, 2.5 and 3.2 times per year for groups A, B, C and D, respectively. The Kruskal-Wallis test provided evidence of an association between deworming frequency and risk category in dogs, this frequency being significantly lower for risk group A (Kruskal-Wallis test: *χ*^2^ = 9.0614, *df* = 3, *P* = 0.0285). However, median frequencies were 1, 3, 2.5 and 3 times per year respectively, and thus similar for groups B, C and D. Replies to questions regarding ownersʼ opinions about deworming in dogs were scaled from 1 (“does not apply at all”) to 7 (“fully applies”). Most dog owners returning replies scaled 5 to 7 (80.44%, 403/501) indicated that they believed their current deworming regimen was sufficient. Almost all owners took into account their veterinarianʼs recommendations awarding scales to this question of 5 to 7 (84.83%, 425/501). Some owners agreed with the statement “I worry I will harm the pet if I deworm it more frequently” and 60.48% (303/501) of owners provided replies to this idea scaled from 5 to 7.

The deworming frequency recommended for dogs in risk group A is 1–2 times a year. This recommendation was met by 64% (16/25) of the dogs assigned to this risk group; the mean annual deworming frequency was 2.4 ± 1.6.

The deworming frequency recommended for dogs in risk group B was 4 times a year. A high proportion of dogs in this risk group 76.7% (23/30) were dewormed less often than recommended. The mean annual deworming frequency in this group was 2.7 ± 2.2. The deworming frequency recommended for dogs in risk group C is more than 4 times a year. Roughly half of the dogs in this risk group (54.2%, 19/35) were dewormed less often than recommended. The mean annual deworming frequency was 3.1 ± 1.8). The deworming frequency recommended for dogs in risk group D is once a month. According to the responses, 96.5% (397/411) of dogs in risk group D were dewormed less often than recommended. The mean annual deworming frequency in this group was 3.2 ± 2.3.

No significant difference in risk groups assigned were observed according to whether dogs were kept in cities, suburban areas, towns or rural areas (Kruskal-Wallis test: *χ*^2^ = 4.7973, *df* = 3, *P* = 0.1873). No significant associations were found between the mean annual frequency of deworming and the variables: being under 6 months old (Kruskal-Wallis test: *χ*^2^ = 2.6808, *df* = 1, *P* = 0.1016), having contact with children or elderly persons (Kruskal-Wallis test: *χ*^2^ = 0.0305, *df* = 1, *P* = 0.8614) or living in cities, suburban areas, towns or rural areas (Kruskal-Wallis test: *χ*^2^ = 3.4365, *df* = 2, *P* = 0.3291).

When owners were stratified according to their attitude towards their pets as well with regard to their sources of information on deworming (Table 2), we observed that deworming was less frequent in sceptical pet owners (2.5 ± 1.6) compared to affectionate (3.1 ± 2.0), devoted (3.6 ± 2.9) or dispassionate (3.0 ± 1.9) pet owners (Kruskal-Wallis test: *χ*^2^ = 10.9413, *df* = 2, *P* = 0.0120). With regard to gender, female owners reported a mean annual frequency of deworming of 3.3 ± 2.3, which was significantly higher than reported by men (2.9 ± 2.3) (Kruskal-Wallis test: *χ*^2^ = 9.8990, *df* = 1, *P* = 0.0017). Mean deworming frequencies reported by pensioners (> 65 years-old) were lower (2.7 ± 1.3) than by employees (36–65 years-old; 3.2 ± 2.4) or middle aged persons (18–35 years-old; 3.1 ± 2.0) but the difference lacked significance (Kruskal-Wallis test: *χ*^2^ = 0.5744, *df* = 2, *P* < 0.7504). Dogs spending outdoor time only in their own gardens showed a mean annual deworming frequency (2.6 ± 2.0), which was significantly lower than the frequency reported by the owners of other dogs (3.3 ± 2.3) (Kruskal-Wallis test: *χ*^2^ = 15.8168, *df* = 1, *P* < 0.0001).

### Cats

According to the data reported by owners, 8.80% (44/500) cats were under 6 months-old; and 79% of cats had contact with children or elderly persons. A large proportion of cats, 70.80% (354/500), were kept indoors permanently. Among the cats free to go outdoors, 63.01% hunted and 63.70% of them caught prey (mice, insects, bats etc.). Of the indoor cats or cats that did not catch prey, 5.90% were given raw meat to eat. In summary, 17 cats lived indoors and ate raw meat, 12 of which lived with children. Details are provided in Table 3.

According to Spanish ESCCAP guidelines, practically all cats were assigned to the minimum risk of parasite infection category A (indoor cats, 62%, 310/500) or to the maximum risk group D (outdoor cats, 32.8%, 164/500). Only 3.4% (17/500) and 1.8% (9/500) of cats were assigned to groups B and C, respectively. According to owners, the mean annual deworming frequency in cats was 2.56 ± 2.17 (Fig. 2). By risk group A, B, C and D, respectively, frequencies were 2.4, 2.4, 2.6 and 2.8 times per year. The Kruskal-Wallis test provided no evidence of an association between deworming frequency and risk category in cats (Kruskal-Wallis test: *χ*^2^ = 2.3908, *df* = 2, P = 0.4954). Moreover, median frequencies were twice per year for every risk category. Replies to questions regarding ownersʼ opinions about deworming in cats were scaled from 1 (“does not apply at all”) to 7 (“fully applies”). A high proportion of cat owners returning replies of 5 to 7 (74.8%, 374/500), indicated they believed their current deworming regimen was sufficient. Almost all owners took into account their veterinarianʼs recommendations awarding scales to this question of 5 to 7 (86.8%, 434/500). Over half the cat owners 59.8% (299/500) agreed with the statement “I worry I will harm the pet if I deworm it more frequently”, reflected by the replies to this concept provided as scales from 5 to 7.

The deworming frequency recommended for cats in risk group A is 1–2 times a year. A small proportion of cats in this risk group (4.6%, 14/304) were not dewormed at all while most (59.9%, 182/304), were adequately treated. The mean annual deworming frequency in this risk group was 2.4 ± 1.9. The deworming frequency recommended for cats in risk group B is 4 times a year, while the mean annual deworming frequency recorded in this group was 2.7 ± 2.3. A high proportion of cats (75.8%, 22/29) were dewormed less often than recommended. The deworming frequency recommended for cats in risk group C is more than 4 times a year, while the mean annual deworming frequency recorded in this group was 2.2 ± 1.2 and a high proportion (83.3%, 25/30) were dewormed less often than recommended. Finally, the deworming frequency recommended for cats in risk group D is 12 times per year, while the mean annual frequency of deworming recorded in this group was 2.9 ± 2.8). In total, 97.1% (133/137) of cats in risk group D were dewormed less often than recommended.

Significantly more cats living in rural areas were assigned to risk group D compared to cats living in cities, suburban areas and towns (Kruskal-Wallis test: *χ*^2^ = 23.0960, *df* = 2, *P* < 0.0001). In this risk group (*n* = 137), no significant associations were found between mean annual deworming frequency and the variables: being under 6 months old (Kruskal-Wallis test: *χ*^2^ = 0.4661, *df* = 1, *P* = 0.4948), having contact with children or the elderly (Kruskal-Wallis test: *χ*^2^ = 2.0835, *df* = 1, *P* = 0.1489) or living in cities, suburban areas, towns or rural areas (Kruskal-Wallis test: *χ*^2^ = 4.4856, *df* = 2, *P* = 0.2136).

When owners were stratified according to their attitude towards their pets as well with regard to their sources of information on deworming (Table 2), we observed that deworming was less frequent deworming in sceptical pet owners (2.0 ± 1.6) compared to affectionate (2.4 ± 1.9), devoted (2.9 ± 2.5) and dispassionate (2.5 ± 2.1) pet owners (Kruskal-Wallis test: *χ*^2^ = 20.1905, *df* = 2, *P* = 0.0002). No significant differences were detected related to owner gender. Mean deworming frequencies reported by pensioners (> 65 years-old) were lower (1.8 ± 1.1) than by employees (36–65 years-old; 2.6 ± 2.2) or middle-aged persons (18–35 years-old; 2.6 ± 2.2) but the difference lacked significance (Kruskal-Wallis test: *χ*^2^ = 2.6715, *df* = 2, *P* < 0.2630). Moreover, cats kept indoors permanently showed a mean annual deworming frequency of 2.4 ± 1.9, significantly lower than the frequency reported by the owners of other cats (2.9 ± 2.7; Kruskal-Wallis test: *χ*^2^ = 4.2959, *df* = 1, *P* = 0.0382).

This survey revealed that the average number of dewormings per year reported in cats was significantly lower compared to that reported in dogs (Kruskal-Wallis test: *χ*^2^ = 34.4386, *df* = 1, *P* < 0.001).

## Discussion

In the present study, we analyzed the deworming frequency reported by Spanish pet owners as part of a large-scale study including European dog and cat owners [31]. If we consider that respondents are likely to be more interested in the health and care of their pets than the average pet owner, responses could be biased.

According to this survey based on Spanish ESCCAP guidelines, a high percentage of the dog population examined (96%) was assigned to the highest-risk endoparasite infection group D, for which monthly deworming treatments are recommended by the ESCCAP. Most cats were indoor cats classified as minimum risk category A (62%). The remaining cats were outdoor cats assigned to the maximum risk category D (32.8%). These risk group D cats mostly lived in country areas rather than towns or villages, which is similar to the situation reported for Germany [32]. More dogs were in the higher risk group than cats, despite being more frequently kept indoors. A high proportion of cats were described to remain indoors 70.80% all the time, while only 20.36% of dogs were restricted to their own gardens and had no contact with public places such as parks, sandpits or playgrounds. Permanently indoor cats showed a mean annual deworming frequency of 2.4, significantly lower than that of the remaining cats (2.9). In dogs with outdoor access only to their garden this frequency was 2.6, significantly lower than that indicated for the other dogs (3.3). Thus, the respective living conditions of cats and dogs gave rise to clear differences in owner deworming patterns [31]. Cat owners did not deworm their pets as often as dog owners (2.56 and 3.13 times per year, respectively), such that deworming practices were consistent with infection risk [33]. Cat owners may deworm their cats less often because several studies have shown a greater risk of parasite infection in outdoor cats [34], yet some authors found the reverse situation [35]. In effect, in a recent study in inner Barcelona, significant prevalence of *D. immitis* was detected in indoor cats precisely because these cats are not usually subjected to preventive measures against parasites [36]. There is also a possibility of reinfection or larvae reactivation in kittens or immunocompromised older cats, such as during pregnancy. Also cats that live with dogs may become infected *via* dog hair contaminated with eggs. Infection may also occur *via* percutaneous route [37] and/or through the ingestion of parasite hosts such as rodents or different insects. Adult cats could then also pose a risk for their owners [38, 39].

Our survey revealed close contact between pets and immunocompromised individuals like children or elderly persons (79% for cats and 90.62% for dogs). This large proportion of pets living with this type of owner supports the frequent use of deworming measures to prevent the spread of zoonotic parasites [31]. To significantly reduce the presence of long-lived *Toxocara* spp. infective eggs in pet faeces and hence in the environment, companion animals need to be dewormed more than four times a year [40, 41].

In dogs, we detected a link between deworming frequency and risk category such that those assigned to group A were dewormed significantly less often. However, deworming frequencies in cats were similar across risk groups. This meant that across every pet risk category, only low-risk dogs were dewormed significantly less often than the remaining pets. As found in other studies, these data indicate that owners deworm their pets regardless of their assumed infection risk [33, 42]. In endemic countries, the less than adequate frequency of deworming determines that dogs and cats are unprotected against a variety of helminths [40].

Based on ESCCAP guidelines, a relatively high percentage of dogs in risk group A (64%, 16/25) were adequately treated, and the rest were treated more often than necessary. A similar scenario was observed for cats in risk group A, in which over half (59.9%, 182/304) were adequately treated, a small proportion (4.6%, 14/304) were not treated, and the remaining cats (35.5%, 108/304) were treated more often than necessary. Thus, pets in the lower risk group A were either adequately dewormed or dewormed more than necessary, with only a small proportion of cats not being dewormed at all (*n* = 14). All pets in group A (except the 14 non-treated cats) carried little risk of worm infection as they were adequately or more frequently treated than recommended. Some cat owners believe that deworming indoor cats is unnecessary [33], although this was not observed much here. According to living conditions and behaviour, more than half of the cats were assigned to the lower risk group A requiring fewer deworming treatments, which matched the ownersʼ reported deworming schedules. In contrast, a high proportion of dogs in risk groups B, C and D (92.2%, 439/476) or cats in these risk groups (91.8%, 180/196) were dewormed less often than recommended. Alarmingly, almost all pets in the remaining groups B, C or D (representing 95% of dogs and 39% of cats) were dewormed less often than recommended. Given the greater proportion of dogs in these higher-risk groups, these pets were more often inadequately treated than cats.

Despite the reported lack of adequate treatment, most pet owners believed that their current deworming regimen was adequate for both dogs and cats (80.44% and 74.8%, respectively). Moreover, almost all owners indicated they relied on their veterinarian’s recommendations (84.83% of dog owners and 86.8% cat owners). In the light of these results, pet owners seem to have a deficient awareness of the zoonotic risks posed by parasites and/or receive insufficient information about this issue from their veterinarians. Extensive literature exists highlighting this owner lack of awareness in relation to zoonoses and their implications for animal and public health [33, 42–45].

In terms of owner attitude towards their pets as well with regard to their sources of information on deworming, we detected less frequent deworming for dog and cat owners who were indifferent about the need for preventive treatment (2.5 and 2.0, respectively) compared to affectionate (3.1 and 2.4 times per year, respectively), devoted (3.6 and 2.9 times per year, respectively) or dispassionate (3.0 and 2.5 times per year, respectively). Therefore, it seems that owners more implicated in their petʼs healthcare tend to deworm their pets more often. In the case of dogs, women showed a significantly higher mean annual deworming frequency (3.3) compared to men (2.9).

In Spain, dogs, especially, show a high risk of helminth infection due to inadequate deworming. In fact, despite the many deworming products available on the market against intestinal parasites [46], the prevalence of intestinal worms in household dogs and cats is within the range of 4.2–83.0%, and *Toxocara* spp. and species of the family Ancylostomatidae have been frequently found [5, 6, 10, 13, 47, 48]. These data point to the need for practitioners to prescribe pet owners a deworming regimen based upon ESCCAP guidelines, and for specific health education designed to improve both animal and public health in accordance with One Health principles [6, 49]. Treatment compliance among pet owners could be improved through reminder methods such as mobile applications and/or through social media [50, 51].

It is the responsibility of veterinarians to educate pet owners about the importance of properly deworming their pets and implementing recommended hygiene measures (e.g. avoiding raw pet food, cleaning pet litter daily). The One Health concept with respect to zoonoses, pets and parasites is clear about why veterinarians, physicians, nurses and public health authorities need to work together to ensure that all decisions and implemented measures have an impact on the health of humans, animals and the environment [52].

## Conclusions

Health education recommendations provided by veterinarians to pet owners is the key to endoparasite control and reducing current parasite prevalences in companion animals. For the management of zoonotic endoparasites, the role of health education (on the part of the owners, veterinarians, physicians, nurses, and health authorities) under the umbrella of the One Health concept is crucial.

## Data Availability

The datasets supporting the conclusions of this article are included within the article. Due to commercial confidentiality, data not included in the manuscript can only be made available to *bona fide* researchers and are subject to a non-disclosure agreement.

## Abbreviations

ESCCAP: European Scientific Counsel Companion Animal Parasites
SD: standard deviation
SE: standard error
